# ALARUM: Active One Health surveillance in LMICs to monitor and predict Antimicrobial Resistance Using Metagenomics – a cross-sectional study protocol

**DOI:** 10.1136/bmjopen-2025-107465

**Published:** 2026-02-24

**Authors:** Marianne A B van der Sande, Daniel Valia, Caroline Tigoi, Nicole Stoesser, Linda Stamm, Andrea Marten, Bram Riems, Robert Musyimi, Yougbare Sibidou, Anita C Schurch, Eric W Tiendrebeogo, Shalton Mwaringa, Malte Kohns Vasconcelos, Brecht Ingelbeen, Halidou Tinto, Julia A Bielicki, Ben S Cooper, James A Berkley, Esther van Kleef

**Affiliations:** 1 Global Public Health and Bioethics, Julius Center, University Medical Centre Utrecht, Utrecht, The Netherlands; 2 Direction Régionale du Centre-Ouest/Clinical Research Unit of Nanoro, Institut de Recherche en Sciences de la Santé, Ouagadougou, Burkina Faso; 3 KEMRI-Wellcome Trust Research Programme, Kilifi, Kenya; 4 Modernising Medical Microbiology, University of Oxford Nuffield Department of Medicine, Oxford, UK; 5 National Institute for Health Research Health Protection Research, Unit Healthcare Associated Infections and Antimicrobial Resistance, University of Oxford, Oxford, UK; 6 National Institute for Health Research Oxford Biomedical Research Centre, Oxford University Hospitals NHS Foundation Trust, John Radcliffe Hospital, Oxford, UK; 7 Paediatric Research Centre, University Children’s Hospital Basel, Basel, Switzerland; 8 Institute of Tropical Medicine Department of Public Health, Antwerp, Belgium; 9 Department Medical Microbiology, University Medical Centre Utrecht, Utrecht, The Netherlands; 10 Institute for Medical Biometry and Epidemiology, University Medical Center Hamburg-Eppendorf, Hamburg, Germany; 11 Department Infectious Disease Epidemiology, Julius Center, University Medical Centre Utrecht, Utrecht, The Netherlands; 12 Institut de Recherche en Sciences de la Sante, Ouagadougou, Burkina Faso; 13 Centre for Neonatal and Paediatric Infection, City St George’s, University of London, London, UK; 14 Nuffield Department of Medicine, Centre for Tropical Medicine and Global Health, University of Oxford, Oxford, UK

**Keywords:** antimicrobial resistance, health demographic surveillance systems, mobile genetic elements, one health surveillance, pooled metagenomics, prediction, sub-Sahara Africa

## Abstract

**Abstract:**

**Background:**

In rural sub-Saharan Africa (sSA), the burden of antimicrobial resistance (AMR) remains high. As AMR continues to rise, there is a strong need for practical, implementable surveillance to monitor and mitigate risks, as well as inform timely, evidence-based clinical decision-making. Emerging evidence points to possible community-level drivers, such as transmission between human, animal and environmental reservoirs as contributing factors, yet microbiological surveillance or opportunities for wastewater-based surveillance are often limited and insufficient in these settings. Therefore, alternative sustainable and affordable approaches are needed. We intend to build on the demonstrated potential of metagenomic profiling of pooled faecal material, which accurately predicted population-level AMR prevalence in invasive *Enterobacterales* infections.

**Methods and analysis:**

We aim to validate this metagenomic pooled approach on additional populations, and to evaluate whether AMR patterns could be similarly predicted from surveillance of community One Health reservoirs. We will assemble existing data from hospital-based microbiology diagnostic laboratories in rural Burkina Faso and Kenya, and determine to what extent community-level metagenomic data, and/or faecal material of patients on hospital admission, can predict AMR in clinical isolates. We will perform community-level surveys in eight clusters per country, randomly selecting 15 households per cluster. We will systematically sample suspected environmental AMR exposure sites in and around households (soil, drinking water, latrines, chicken faeces) and collect data on community-level antibiotic use, hygiene practices, contact with domestic animals and sanitary facilities. Samples and data will be collected twice: during the dry and during the rainy season.

In addition to evaluating the accuracy of predicting resistance in clinical isolates, we will quantify community-level exposure risks. We will conduct metagenomic profiling on pooled DNA extracts from human stool samples (hospital and community-level) and from household environments. Bayesian statistical models will quantify relationships between AMR gene abundance in the environment and in human stool, and invasive bacteria identified among clinical patients, accounting for geography and seasonality. A cost-utility analysis will determine under what circumstances the use of pooled metagenomic data to inform empirical antibiotic policies would represent an efficient use of resources.

**Ethics and dissemination:**

The proposed surveillance protocol is developed in partnership with local communities and local and international researchers and has received ethical approval in Kenya and Burkina Faso. It will assess whether intermittent, pooled-sample metagenomics provides a viable, low-cost and practical approach for population-level AMR surveillance in settings that—like many in rural sSA—lack systematic microbiological diagnostics and where sewage systems for wastewater-based surveillance are absent. By providing an alternative to routine microbiological-based surveillance where this proves challenging to implement, this approach may help improve treatment outcomes, contribute to equity and public health. Findings will be disseminated through peer-reviewed publications and academic conferences and will contribute to the recently proposed WHO AMR surveillance strategy, which combines survey-based approaches with routine AMR surveillance.

**STRENGTHS AND LIMITATIONS OF THIS STUDY**

- Build on long-standing community collaborations, emphasising equity.
- Develop an innovative approach to antimicrobial resistance (AMR) surveillance in line with recent WHO recommendations.
- Explore metagenomics on pooled samples as an alternative to culture single sample-based One Health AMR surveillance.
- Strengthen the capacity of field and laboratory staff, including epidemiological studies, molecular microbiological work, bioinformatic processing and interpretation of genomic One Health data.
- Scalability of methods will need awareness of and sufficient resources for controlling AMR.

## Introduction

 Antimicrobial resistance (AMR) is a global health emergency, impacting health and socioeconomic development, in particular in low-resource settings.^1^ While surveillance data remain limited in many low- and middle-income countries (LMIC), resistance prevalence rates of over 80% against commonly used antibiotics have been reported in clinical settings in sub-Saharan Africa (sSA).^2 3^ Timely and robust surveillance data are essential to support clinical decision making, monitor emerging AMR risks and guide targeted interventions.

In sSA, public hospitals often lack reliable microbiological laboratories, limiting AMR surveillance to a few tertiary teaching hospitals or research institutions.^4 5^ Surveillance reports generally do not distinguish between community-acquired and hospital-acquired infections, which are likely to have different AMR patterns, reducing their utility for guiding treatment.^6^ The low rates of blood culture utilisation in sSA have resulted in large biases in the estimated proportion and incidence of AMR infections.^6^ Delays in healthcare seeking, prior antibiotic use in communities where most initial treatment is carried out by dispensaries and health centres or self-medication, and a variable testing remit, also mean that surveillance data from LMICs—where it exists—often fail to reflect true AMR prevalences in community-acquired infections.

One Health surveillance approaches involving environmental reservoirs such as urban sewage, slaughterhouse waste or manure have been proposed as a convenient and cost-efficient approach to monitor AMR emergence and trends in communities^7 8^ or to assess exposure and transmission risks of AMR across One Health sectors.^9 10^ The relevance of such data for timely awareness of AMR patterns in clinical infections, however, remains unclear. Additionally, the absence of formal sewage infrastructure and the predominance of small-scale farming make classical wastewater-based or livestock farm-based surveillance approaches largely unfeasible in typical rural sSA settings.

Recent metagenomic studies across global One Health environments have shown that nearly a quarter of identified antimicrobial resistance genes (ARGs) may pose a risk to human health.^11^ Interestingly, around 90% of these ARGs appear to be confined to specific environmental domains—such as aquatic, terrestrial or built environments. This may suggest substantial variation shaped by local ecological and infrastructural factors, including sanitation systems, proximity to animals, antibiotic usage patterns and healthcare exposure. These estimates are from studies focusing predominantly on marine environments, with limited data from terrestrial sSA, where AMR dynamics may be influenced by very different conditions.^12^

In parallel, there is growing interest in how metagenomic surveillance can be applied in clinical settings. A study by Tosas Auguet *et al*^13^ demonstrated that pooled faecal metagenomic profiling at hospital admission could accurately predict AMR prevalence in Enterobacterales clinical isolates across 16 antibiotics in Cambodia, Kenya and the UK. This approach—low-cost and population-based—holds particular promise for guiding empiric treatment decisions in settings where quality-assured microbiological diagnostics remain unavailable. So far, its application has been limited to hospital-based surveillance, but expanding this strategy to include environmental and community compartments has yet to be explored, whereby samples can be sent to a central national laboratory for analysis. Doing so offers a dual benefit: helping tailor empiric antibiotic guidance at the local level, while also identifying key exposure risks and AMR drivers across One Health interfaces. Taken together, this integrated metagenomic approach could support both clinical and public health decision-making in regions with limited diagnostic infrastructure. We aim to develop and test this novel methodology for low-cost One Health sentinel AMR surveillance.

## Methods and analysis

**Figure 1:**
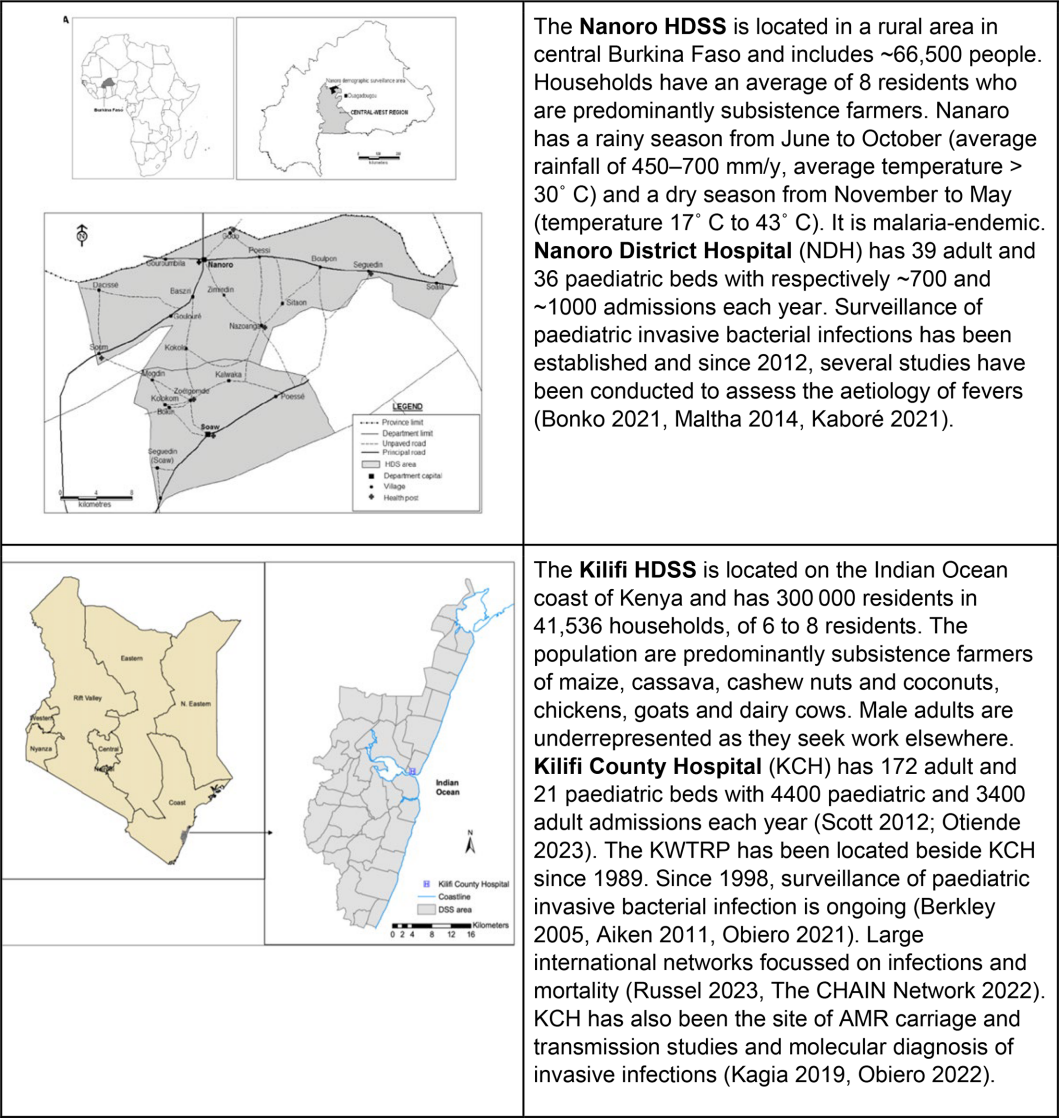
Burkina Faso and Kenya study sites. AMR, antimicrobial resistance; HDSS, Health Demographic Surveillance Systems; KWTRP, KEMRI/Wellcome Trust Research Programme.

We will build on existing collaborations with well-established long-term Health Demographic Surveillance Systems (HDSS) in Burkina Faso (Nanoro) and Kenya (Kilifi)^14 15^ (figure 1).

**Figure 2:**
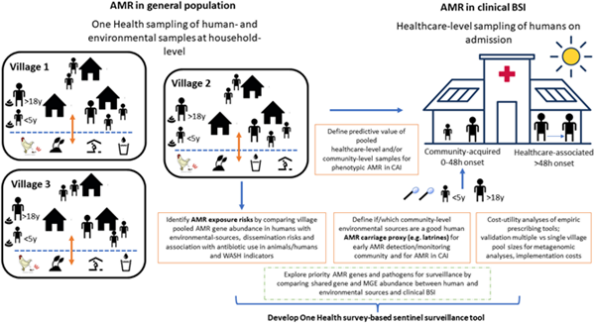
Building towards survey-based One Health sentinel surveillance. AMR, antimicrobial resistance; BSI, Bloodstream Infection; CAI, community acquired infection;MGE, mobile genetic elements; WASH, water, sanitation and hygiene.

To develop and test a survey-based sentinel surveillance approach, we will undertake community and hospital-based surveys including human and environmental sampling to test the performance of community samples at different locations in predicting AMR in invasive isolates at the hospital level. We will test this using the existing clinical AMR surveillance. Blood culture results from patients admitted with suspected bloodstream infections will serve as reference data to assess which population-level metagenomics approach most accurately predicts susceptibility patterns in invasive disease. Additionally, we will attribute environmental and livestock resistomes to pooled human resistome profiles, as well as examine how these pathways relate to antibiotic use and Water, Sanitation and Hygiene (WASH) practices, to pinpoint dominant exposure routes and AMR drivers across One Health interfaces. This leverages established AMR research capacity in both sites^16 17^ (figure 2).

### Data collection

#### Hospitals

In each hospital setting, we will enrol 200 children aged <5 years, and 200 adults ≥18 years; half in the dry season, half in the wet season for rectal sampling. Inclusion criteria will be admission with an acute illness from a household location which falls within the community HDSS area. Exclusion criteria will be a transfer from another hospital, hospital department or maternity ward, admission for trauma or elective surgery or lack of consent. Background demographic, epidemiological and clinical patient data, as well as clinical and microbiology data on hospital admission will be collected, including speciation and susceptibility testing results of any bacterial pathogens identified. Reported or documented prehospital antimicrobial use will be collected at admission. We will also collect data on prior visits to health centres, hospitals (within <1 week, <1 month and <6 months before this admission) and self-medication (over the counter from pharmacies or medicine vendors) for the index patient. Based on symptoms, clinical signs and diagnostic tests, a main clinical presentation will be determined for each participant, based on clinical presentations in WHO’s AWaRe Antibiotic book.^18^

To estimate hospital antibiotic use, data on prescribed antibiotics will be collected from all inpatients present in selected wards on specific days, using the standardised Global inpatient point prevalence survey (PPS) for AMR and consumption.^19^ In line with this protocol, detailed information on patients and antimicrobial use from patients receiving at least one antimicrobial on the day of the PPS and denominator data will be collected at ward level.

#### Communities

In both Burkina Faso and Kenya, eight villages will be purposely selected from within the areas covered by each HDSS in Nanoro and Kilifi respectively. Selection will be made from the HDSS catchment areas, following geographical stratification to ensure representing the range of population, geographical and environmental diversity. Household Global Positioning System (GPS) coordinates will be available in both Burkina Faso and Kenya. Only villages with at least one provider of antibiotics are eligible.

In each community cluster, 15 households will be randomly selected. Following informed consent, in each household, data and stool samples will be collected (max 6 stool/household, randomly selected if more people are present), giving an expected total of around 75 stool samples/village. Indicators of population risk of within-household AMR exposure and dissemination, including hygiene practices, contact with domestic animals, sanitary facilities, recent hospitalisations and medicine use, will be equally assessed in each household. Next, we will sample suspected environmental AMR exposure sites in and around the 15 households per village cluster. We intend to sample four different potential environmental reservoirs per household: chicken faeces (also a proposed sample reservoir in the WHO’s Tricycle protocol),^9^ latrines or open-air defecation areas, drinking water sources and soil, expecting 60 environmental samples per village in total (slightly fewer if certain environmental reservoirs are shared between households).

To quantify community antibiotic use, patient exit interviews will be conducted in the wet and dry season among patients completing a visit to a medicine provider.^20^ We will use a structured questionnaire to document symptoms, quantity of antibiotics dispensed/purchased by antibiotic group if any and if applicable, dose and duration of antibiotic treatment (including any increase/decrease), method of administration, number of antibiotics and antimalarials used concomitantly. A photograph of the antibiotic and its packaging will be taken using the mobile data collection devices. Medicine providers will be asked for their agreement to participate in the survey, and informed consent will be sought from each adult patient, or from caretakers of minor patients (in addition to verbal assent of adolescent patients) to participate in the survey.

#### Metagenomic sequencing and profiling

The optimal minimum metagenome sequencing depth will be defined using available metagenomes.^13 21^ Then, optimal DNA extraction strategies will be explored, comparing individual DNA extractions followed by pooling of extracts, versus pooling samples prior to DNA extraction. This will be done by collecting the rectal swabs of hospitalised patients in duplicate, using one swab for the individual extraction, the other swab for pre-extraction pooling. Pre-extraction pooling will combine 67 samples, stratified by age group, resulting in three adult pools and three paediatric pools per country. From the individual extractions in the hospital and the community, metagenomes will be created from various strata of patients, healthy community members and environmental samples.

For each predefined sample grouping, metagenomic DNA will be extracted from samples using a commercial kit (eg, PowerSoil DNA isolation kit (Qiagen)). Metagenomic sequencing of these pools will take place on an Illumina NovaSeq 6000 to maximise data yield per unit cost, and to a depth up to a maximum of 55 Gb data.^13^ Bacterial species abundances and the ‘resistome’ will be characterised using a metagenomics data processing pipeline. This will use validated species taxonomic profilers (ie, Kraken, Bracken) to estimate bacterial species abundances, and AMR gene profilers (eg, RGI-bwt) to define presence or absence of any given AMR gene allele, based on mapping depth and reference allele lateral coverage thresholds. We will use these outputs to map and aggregate counts of AMR genes/variants associated with resistance to specific antibiotics, adjusted for abundance of pathogens of interest (eg, *Enterobacterales*).

Recent advances in strain haplotyping algorithms also enable the exploration of bacterial strain diversity directly from short-read metagenomic data, without requiring culture of individual isolates, allowing dominant strain variants to be characterised and cross-compared between reservoirs.^22^

To examine the genomic context of key AMR genes such as those in the Extended-Spectrum Beta-Lactamase (ESBL) group, we will use tools like MetaMobilePicker to screen metagenomic assemblies for co-occurrence of AMR genes and mobile genetic elements (MGE), acknowledging the limitations of short-read data in metagenomic assembly. The assembly-based analysis of MGEs will allow analysis of the genetic context of ARGs, leading to a prediction of mobility of ARGs (eg, being located on plasmid) which, in turn, will lead to a prediction of mode of transfer of ARGs between bacterial isolates (eg, clonal vs horizontal dissemination). We will apply kernel-penalised regression modelling to assess associations between household-level and village-level factors and resistome composition in community samples.

### Statistical analysis

#### Exploration of One Health AMR exposure pathways

We will generate a One Health understanding of the dynamics of AMR in rural African communities to inform AMR trends and the design and evaluation of integrated One Health interventions. Exploratory analysis will aim to characterise important patterns in the metagenomic data, including species, MGEs and ARG patterns, allowing us to explore the degree of similarity in species, MGE and ARG profiles across different geographical areas and One Health settings. We will employ maximisation-based attribution methods such as fastST, SourceTracker2 and FEAST to quantify source attribution of strains being shared among different types of microbiomes to estimate the proportional attribution of sampled household resistomes (eg, in environments and among livestock) to pooled human resistome profiles. These approaches infer compositional similarity rather than direct gene transfer but can identify likely environmental or animal reservoirs contributing to AMR patterns identified in human stool. Subsequently, we will apply kernel-penalised regression modelling to assess associations between household-level and village-level factors and resistome composition in community samples. This analysis will provide important insights about ecological interactions between One Health compartments and inform a mechanistic understanding of the processes involved.

#### Predicting AMR patterns in clinical isolates

**Figure 3:**
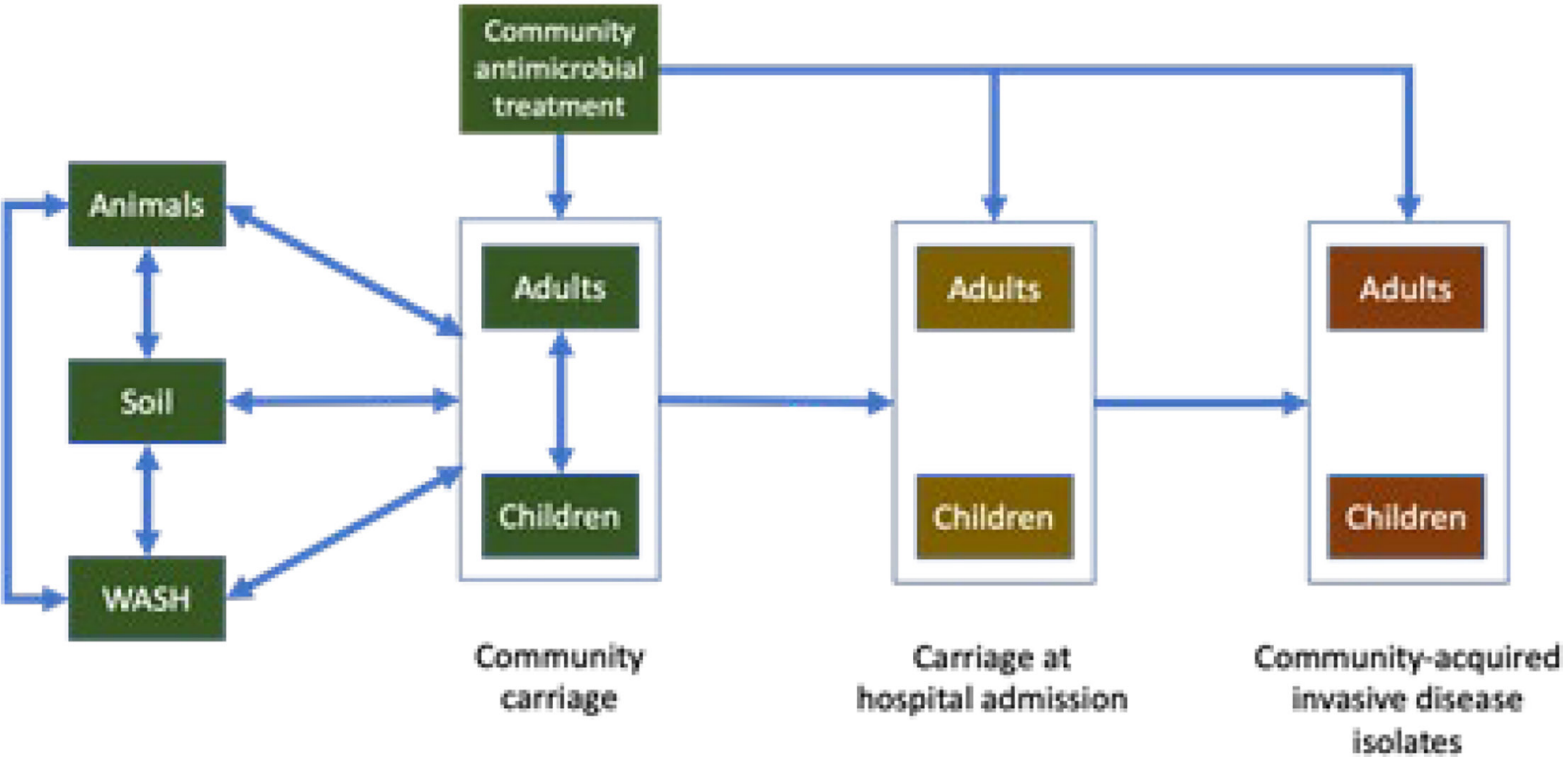
Conceptual framework of the linkage of One Health compartments and settings in an Active OH surveillance approach.

We will determine the utility of pooled-sample metagenomic One Health surveillance targeting each of the One Health compartments for predicting clinically important AMR in hospital bloodstream bacterial isolates as observed in routine surveillance and informing empiric prescribing (figure 3).

Here, predictive modelling will evaluate different taxonomy-adjusted AMR metrics and risk-adjusted AMR metrics from population-pool metagenomics data to test their potential to predict the numbers of invasive infections that are resistant to a given antibiotic in local hospital data. We will also develop and evaluate models for predicting patterns of resistance to multiple antibiotics in individual clinical isolates. Our design allows us to relate the carriage-based metagenomic resistome data to phenotypic resistance observed in invasive isolates from the same settings. We will compare the predictive performance of AMR models based on metagenomic data from (pooled) community samples of healthy individuals and from household environments with those derived from hospital-admitted febrile patients, and models using these combined data. The predictive value of three groups of models will be compared: (1) those that make use of resistance metrics derived only from human stool samples; (2) those that make use of resistance metrics derived only from household environment samples; (3) those that make use of metrics derived from both human and environmental samples. Baseline models including only resistance gene abundance metrics, only taxonomic metrics and an intercept-only null model will also be assessed to determine the added value of combining taxonomic and AMR data.

To account for potential seasonal variation in gene abundance and subsequent AMR resistance patterns in clinical isolates, we will first describe seasonal differences in metagenomic resistome composition and abundance metrics. Next, we will include season (wet vs dry) as a covariate in the Bayesian hierarchical binomial models used to predict clinical resistance, and test for interaction between season and metagenomic predictors to assess whether associations differ by season. We will fit season and non-season stratified Bayesian models to evaluate whether model coefficients and predictive accuracy (based on leave-one-out cross-validation) differ between data sources used, gene abundance metrics and seasons. Posterior predictive checks will confirm that the models capture observed AMR patterns in clinical samples. Environmental covariates such as rainfall will be incorporated where available.

### Cost-utility of the One Health sentinel surveillance approach

An economic analysis will explicitly consider trade-offs between the benefits of increased prediction accuracy associated with larger sample sizes and decreased pooling, and the additional costs involved. We will quantify the expected benefits of antibiotic policies informed by pooled sample metagenomic surveillance data in terms of life years and quality adjusted life years gained compared with the status quo of no local microbiological surveillance. We will use simulations to explore how cost-utility varies with different sampling strategies in the surveillance data (including varying environmental sample pool sizes), varying AMR prevalence and the degree of geographical heterogeneity within the population of interest.

### Sample size considerations

In a proof-of-concept study,^13^ metagenomes generated from pooled DNA extracts of rectal swabs/faecal samples of 177, 157 and 156 individuals in Kenya, the UK and Cambodia respectively were used to model AMR prevalence in clinical *Enterobacterales* isolates, resulting in good predictive performance. Pooling will be done across reservoirs, that is, pooling samples from each environmental source and from healthy humans by village, age and season; pooling hospital samples by site, age and season. We will also test to what extent smaller pool sizes would enable accurate predictions and evaluate different pooling formats (by age, by village, by season, by reservoir).

### Data management

A data management plan has been drafted and agreed by all partners, based on the University Medical Centre (UMC) Utrecht template with DPIA V.2.1, detailing all important aspects regarding data management, data protection and access to data. Project data will be shared between consortium members on a need-to-know basis. Anonymisation or at least pseudonymisation will be done at the earliest opportunity and as much as possible. No participant identifiable data will be disclosed from study sites/partners, nor in study databases, analysis and reports. User roles and access controls for study sensitive data will be implemented. Data storage will be done on centrally secured and protected servers or clouds, with secure (encrypted) transfers of study confidential data if needed. Contractual confidentiality agreements will be in place for all involved study staff. Data processing duties and responsibilities will be clearly defined and documented.

### Patient and public involvement

The surveillance to be developed and tested is based in the community and among community members presenting to hospital. Patients are only involved if they consent to be sampled on admission. This project builds on long-standing collaborations with the communities of Nanoro and Kilifi, who actively participate in shaping the research conducted with and in the communities, including for this novel AMR surveillance project. Rationale and design of the study have been finalised in close collaboration with community representatives, who were also involved in previous AMR-related projects, such as the CHAIN study in Kilifi^23^ and the CABU-EICO (Optimising Community AntiBiotic Use and infection prevention and control through bEhavioural Interventions in rural Burkina Faso and DR COngo) study in Nanoro.^16^ As with any study in these HDSSs, regular exchanges with communities take place for dissemination, mutual feedback and input.

## Ethics and dissemination

The project, including informed consent procedures, was approved by the Ethics Committee for Health Research in Burkina Faso (2024-05-137), the KEMRI Scientific and Ethical Review Board (KEMRI/RD/22) and the Institutional Review Board of the Institute of Tropical Medicine Antwerp (1763/24, 1764/24). Individual informed consent is obtained for the exit interviews and for the patients sampled in hospital. For the communities, all included households are active participants in the HDSS, and as such have consented to be approached for further research. For this study, first oral consenting of communities via traditional structures was conducted, followed by consenting of individual household heads.

Our project aims to develop and evaluate a novel, sustainable, low-cost sentinel survey approach enabling One-Health AMR surveillance applicable in low-resource settings. This metagenome-informed surveillance approach incorporating intermittent population-level rather than continuous individual-level sampling aims to address a major gap in understanding and addressing local and global challenges related to control of an increasing AMR threat. If successful, such a framework could be scaled up, leveraging central laboratory sequencing capacity using a ‘hub and spoke’ sampling and sample processing structure and integrating available human disease AMR data to simplify national data collection for policy and guideline implementation. Feedback to and dialogue with local communities, public health and veterinary agencies and hospitals is a key aspect to enable this metagenomics-informed surveillance framework to provide ‘information for action’.

WHO has recently updated its global AMR surveillance strategy by supporting national periodic representative human AMR surveys to supplement the paucity of routine surveillance data in low-resource settings.^24^ If our survey-based AMR metrics are predictive of AMR in clinical infections, countries and hospital sites lacking resources for routine clinical bacterial surveillance could use our metagenomic informed community AMR framework in the future, enabling them to estimate AMR prevalence and patterns in clinical isolates. This could serve to guide empirical treatment selection, based on expected coverage offered by different regimens, potentially improving clinical outcomes. Furthermore, it will enable the monitoring of the emergence of and trends in AMR and identify where interventions would be most needed. If successful, the proposed alternative AMR metrics could be integrated into WHO’s planned scale-up of national representative surveys, increasing the granularity and coverage of surveillance data. For scaling up, we intend to involve first national partners and in a later phase partners in regional networks in East and West Africa. We will seek to identify relevant regional partners with this capacity in Africa and work with existing initiatives present in the region, including Pathogen Watch (developed by the Centre for Genomic Pathogen Surveillance in partnership with University of Oxford and Wellcome Sanger Institute) and African CDC Pathogen Genomics Initiative. Hence, we would not only improve clinical prescribing and situational awareness locally, but also contribute to increasing representativeness of the existing burden of AMR estimates regionally and globally, notably including more and better data from LMIC.

This project will furthermore strengthen South-South collaborations. With our project, we bring together existing AMR surveillance networks based in rural West Africa (Burkina Faso) and East Africa (Kenya), while benefiting from long-standing collaborations with European partners. This will strengthen diagnostic and epidemiological capacity in establishing, analysing and disseminating in a timely fashion relevant data for local and global policymakers, clinicians, stewardship programmes, syndromic treatment guideline committees, public health officials and researchers. Additional training in sampling processing will be provided by the University Children’s Hospital in Basel, the Institute for Tropical Medicine in Antwerp, UMC Utrecht and the University of Oxford. This training will be face-to-face and online and will include validation of Standard Operating Procedures (SOPs), data validation and bioinformatics analysis for metagenomic data. Materials will address both clinical and laboratory staff audiences, providing insights into the importance of preanalytics and discussing key aspects of workflow for research laboratories. Knowledge transfer between institutes is key to the project, and has been embedded in several joint preparatory workshops and regular consortium meetings. Once the sequencing data are generated, a bioinformatics carpentry workshop is planned.^25^

Overall, therefore, the strengths of this project are that it builds on existing scientific collaborations, established community trust and frameworks for knowledge exchange, and pilot data supporting the potential of the innovative metagenomic pooling and AMR surveillance approach. In addition, it addresses a highly relevant global need for sustainable AMR surveillance in low-resource settings, given that the implementation of routine, individual-level diagnostics in these settings remains challenging. Dissemination will occur through community meetings, AMR awareness sessions, peer-reviewed publications and academic conferences.
