# Supplementary material for: ALARUM: Active One Health surveillance in LMICs to monitor and predict Antimicrobial Resistance Using Metagenomics – a cross-sectional study protocol
Source: BMJ Open. 2026 Feb 24;16(2):e107465. doi: 10.1136/bmjopen-2025-107465 (PMC12933788; doi:10.1136/bmjopen-2025-107465)
Supplement: Supplementary file 1 [file bmjopen-2025-107465.reviewer_comments.pdf]

PEER REVIEW HISTORY

BMJ Open publishes all reviews undertaken for accepted manuscripts. Reviewers are asked to complete a checklist review form and are provided with free text boxes to elaborate on their assessment. These free text comments are reproduced below.

ARTICLE DETAILS

Title (Provisional)

ALARUM: Active One Health surveillance in LMICs to monitor and predict Antimicrobial Resistance Using Metagenomics; study protocol: a cross-sectional study

Authors

van der Sande, Marianne AB; Valia, Daniel; Tigoi, Caroline; Stoesser, Nicole; Stamm, Linda; Marten, Andrea; Riems, Bram; Musyimi, Robert; Sibidou, Yougbare; Schurch, Anita C; TIENDREBEOGO, ERIC WENDPOUIRE; Mwaringa, Shalton; Kohns Vasconcelos, Malte; Ingelbeen, Brecht; Tinto, Halidou; Bielicki , Julia Anna; Cooper, Ben S; Berkley, James A; van Kleef, Esther

---

VERSION 1 - REVIEW

**Reviewer**	**1**
**Name**	**Prasad, Ramakrishna**
**Affiliation**	**Academy of Family Physicians of India**
**Date**	**03-Oct-2025**
**COI**	**None**

The protocol submitted is very interesting and likely to be of high value to the scientific community across AMR, OneHealth and public health realms. Some comments:

a) I think a little more detail is required in how you intend to extend the information from looking at similarities of ARGs across various domains to a mechanistic understanding of the processes involved- this seems to me to be over-stretching the interpretation.

b) It would also be useful if you could explain a little more about the models used to predictive analyses (from population pool to invasive infections) - mere presence of ARGs does not imply either phenotypic resistance or potential to cause serious invasive infections.

c) Among the hospital -admitted patients, are there specific infections that you will consider or will you be taking the history, clinical and microbiological information from anyone getting admitted with an infectious cause? What about non-infectious causes? If your imagined mechanism is environment- to - humans - to -environment, then maybe the most obvious

set of organs would be diseases of the skin and urinary tract. By that token, agree that stool specimens are a good set of samples to collect.

d) I notice you are not checking for antibiotic levels in the environmental samples collected. Any particular reason why? Wouldn't that help you corroborate your environmental ARG levels with antibiotic pressure?

e) Would there be a financial or scientific benefit in doing cultures on the environmental samples (on differential/selective media at different dilutions) and collecting specific isolates for meta genomic analyses? That way you would bypass the phenotypic-genotypic differences in resistance profiles of isolates. Would also help strengthen your case with clinicians who would be willing to give more credence to genotypic data associated with live bacteria than just DNA from various sectors.

**Reviewer**	**2**
**Name**	**Kushugulova, Almagul**
**Affiliation**	**Nazarbayev University, National Laboratory Astana**
**Date**	**12-Oct-2025**
**COI**	**None**

This is an important and innovative protocol. It combines metagenomic methods with human, animal, and environmental sampling to improve AMR surveillance in LMICs. The topic is highly relevant to global health and fits well within the One Health approach. Strong scientific rationale and alignment with WHO priorities. Builds research capacity in Africa and promotes equal collaboration. Combines microbiology, genomics, and epidemiology effectively. The background is clear and well supported by recent literature. The goal to create a predictive model for AMR is timely and valuable.

Minor issue: The authors could briefly discuss how this approach could be scaled up and applied in other countries.

Methodology: The methods are comprehensive and detailed, but some points need clarification.

Major comments:

Pooling and sequencing: Please explain how pooling will be done and how you will keep enough biological diversity to avoid missing rare ARGs.

Validation: Describe how metagenomic data will be compared with hospital isolates and what measures (e.g., correlation, AUC) will be used to test accuracy.

Sampling: Clarify how villages were chosen and whether this selection ensures diverse settings.

AMR gene interpretation: Briefly describe how you will link ARGs to their host organisms or functions to improve biological meaning.

Questions for the Authors

How will differences between metagenomic and culture-based AMR results be interpreted?

Will host or immune-related data be collected for context?

What controls will ensure quality and reproducibility between labs?

How will seasonal changes affect AMR results and how will this be analyzed?

How will sequencing cost and pool size be optimized for future use?

Minor to Moderate Revision

This is a high-quality and promising study protocol. After clarifying key methodological points and improving a few sections on data handling and interpretation, it will make a strong contribution to AMR research and the One Health field.

VERSION 1 - AUTHOR RESPONSE

Reviewer 1:

The protocol submitted is very interesting and likely to be of high value to the scientific community across AMR, OneHealth and public health realms.

Thank you for your encouraging feedback, and your specific suggestions.

Some comments:
a) I think a little more detail is required in how you intend to extend the information from looking at similarities of ARGs across various domains to a mechanistic understanding of the processes involved- this seems to me to be over-stretching the interpretation.

Response: The assembly-based analysis of mobile genetic elements with MetaMobilePicker will allow analysis of the genetic context of ARGs, leading to a prediction of mobility of ARGs (e.g. being located on plasmid) which, in turn, will lead to a prediction of mode of transfer of ARGs between bacterial isolates (e.g. clonal vs horizontal dissemination). However, we agree that similarities in ARG across domains need to be analysed carefully, and that we should not jump to conclusions regarding the mechanistic processes underlying this.

To further clarify our planned approaches, we will employ and expand maximisation methods such as fastST (Choi 2025), SourceTracker2 (GitHub - caporaso-lab/sourcetracker2: SourceTracker2) and FEAST (Shenav 2019) to quantify source attribution of strains being shared amongst different types of microbiomes to estimate the proportional attribution of sampled household resistomes (e.g in environments and amongst livestock) to pooled human resistome profiles. These approaches infer compositional similarity rather than direct gene transfer but can identify likely environmental or animal reservoirs contributing to AMR patterns identified in human stool. Recent advances in strain haplotyping algorithms such as Floria also enable the exploration of bacterial strain diversity directly from short-read metagenomic data, without

requiring culture of individual isolates, allowing dominant strain variants to be characterised and cross-compared between reservoirs (ref 23, Kim 2024).

To examine the genomic context of key AMR genes such as those in the ESBL group, we will use tools like MetaMobilePicker to screen metagenomic assemblies for co-occurrence of AMR genes and mobile genetic elements, acknowledging the limitations of short-read data in metagenomic assembly. Finally, we will apply kernel-penalised regression modelling to assess associations between household- and village-level factors and resistome composition in community samples. Combined, these analyses will provide insight into which reservoirs and exposures are most relevant to human AMR patterns across One Health settings.

We have now subdivided the 'statistical analyses' section in '*Exploration of One Health AMR exposure pathways*' and ´*Predicting AMR patterns in clinical isolates'* and added the above explanation on page 8.

Furthermore, in the 'metagenomics and profiling' section, page 7, we detailed our planned MetaMobilePicker analyses.

b) It would also be useful if you could explain a little more about the models used to predictive analyses (from population pool to invasive infections) - mere presence of ARGs does not imply either phenotypic resistance or potential to cause serious invasive infections.

Response: Predictive modelling will adopt a similar methodology to previous work (i.e. Bayesian hierarchical generalised linear models with binomial likelihoods, ref 14, Tosas-Auguet 2021). We will evaluate different taxonomy-adjusted AMR metrics and risk-adjusted AMR metrics from population-pool metagenomics data to test their potential to predict the numbers of invasive infections (bloodstream infections) that are resistant to a given antibiotic in local hospital data from both sites. To ensure reproducibility and methodological validity following deprecation of the original ResPipe workflow, we will explore updated bioinformatics pipelines for resistome profiling, including RGI-bwt and AMRScan (the in-development successor to ResPipe), both based on the CARD database, with Kraken2 used for taxonomic profiling. Comparative analyses will be undertaken to assess consistency of outputs across tools and database versions before integration into the modelling. We will extend previous work by also developing and evaluating models (ref 14) for predicting patterns of resistance to multiple antibiotics in individual clinical isolates. We will compare the predictive value of three groups of models: 1) those that make use of resistance metrics derived only from human stool samples; 2) those that make use of resistance metrics derived only from household environment samples; 3) those that make use of metrics derived from both human and environmental samples. We will also use these earlier datasets to evaluate whether such taxonomy-adjusted AMR metrics and predictive modelling could be extended to non-Enterobacterales pathogens that are known to be gastrointestinal tract colonisers and also important clinical pathogens, such as *Staphylococcus aureus* (ref Marshall 2014) and Enterococcus spp and WHO priority pathogens (ref 2, WHO 2025). Full details of the proposed analytical pipeline are described in Tosas-Auguet et al (ref 14).

We have added this information on page 8.

c) Among the hospital -admitted patients, are there specific infections that you will consider or will you be taking the history, clinical and microbiological information from anyone getting admitted with an infectious cause? What about non-infectious causes? If your imagined

mechanism is environment- to - humans - to -environment, then maybe the most obvious set of organs would be diseases of the skin and urinary tract. By that token, agree that stool specimens are a good set of samples to collect.

Response: To validate the potential of pooled metagenomic sequencing for AMR surveillance in settings where routine clinical microbiology is limited, our predictive models will focus on estimating the numbers of invasive infections (bloodstream infections) resistant to specific antibiotics, as identified through routine clinical surveillance already in place at our two study sites, and detailed in Tosas Auguet et al (ref 14). Among hospital-admitted patients, we therefore include cases with acute febrile illness, from whom rectal swabs are collected on admission. This design allows us to relate carriage-based metagenomic resistome data to phenotypic resistance observed in invasive isolates from the same settings. We will then compare the predictive performance of AMR models based on metagenomic data from (pooled) community samples of healthy individuals and household environments with those derived from hospital-admitted febrile patients. We have added a further description in the statistical analyses section on page 8.

d) I notice you are not checking for antibiotic levels in the environmental samples collected. Any particular reason why? Wouldn't that help you corroborate your environmental ARG levels with antibiotic pressure?

Response: It is correct that we are not measuring antibiotic levels in the environmental samples collected. Interpreting environmental antibiotic concentrations is challenging, as levels can fluctuate, i.e. are transient, and may not reach biologically relevant thresholds for selection. Earlier studies (e.g. DOI: 10.1016/S1473-3099(12)70317-1) note that environmental residues are frequently present at sub-inhibitory concentrations, and direct evidence linking these levels to resistance emergence in situ remains limited. In addition, methods to characterise antimicrobials and their metabolites such as HPLC are expensive, and beyond the scope of the funded project. While such measurements could provide context on continuous AMR selective pressures, and thus could help understand AMR patterns in the community, we argue that this is not essential for our primary objective of predicting AMR in bloodstream infections.

e) Would there be a financial or scientific benefit in doing cultures on the environmental samples (on differential/selective media at different dilutions) and collecting specific isolates for meta genomic analyses? That way you would bypass the phenotypic-genotypic differences in resistance profiles of isolates. Would also help strengthen your case with clinicians who would be willing to give more credence to genotypic data associated with live bacteria than just DNA from various sectors.

Response: Indeed, adding cultures on selective media could be a relevant addition to understand phenotypic AMR. However, as we explained in the introduction, currently, culture-based AMR diagnostics is not widely available in many LMIC, which is why we intend to test the effectiveness of pooled metagenomics as an alternative, which proofed successful in our early pilot work (ref 14). Furthermore, in the selected settings, routine microbiology is challenging, and culturing environmental samples (for a number of clinically important bacteria) comes with inherent difficulties. However, we are planning a source attribution sub-study whereby we will culture stools for *E. coli*, followed by genomic analysis. This will also allow to validate the use of

metagenomic data versus culture data to predict clinical *E. coli* infections from environmental and human samples, along the methodology proposed by the reviewer (see also ref 23 Kim et al). We included this information in the metagenomics section on page 7.

Reviewer 2

This is an important and innovative protocol. It combines metagenomic methods with human, animal, and environmental sampling to improve AMR surveillance in LMICs. The topic is highly relevant to global health and fits well within the One Health approach. Strong scientific rationale and alignment with WHO priorities. Builds research capacity in Africa and promotes equal collaboration. Combines microbiology, genomics, and epidemiology effectively. The background is clear and well supported by recent literature. The goal to create a predictive model for AMR is timely and valuable.

Minor to Moderate Revision
This is a high-quality and promising study protocol. After clarifying key methodological points and improving a few sections on data handling and interpretation, it will make a strong contribution to AMR research and the One Health field.

Thank you for your positive endorsement of our protocol! We addressed your comments and suggestions as follows:
a) The authors could briefly discuss how this approach could be scaled up and applied in other countries.

Response: We added a brief discussion how the approach could be scaled up (page 11). Specifically, we intend to involve first national partners (e.g. the Ministry of Health in Kenya and Ministre de la Santé et de l'Hygiène Publique in Burkina Faso) and in a later phase partners in regional networks in East and West Africa. We will seek to identify relevant regional partners with this capacity in Africa, and work with existing initiatives present in the region, including Pathogen Watch (developed by the Centre for Genomic Pathogen Surveillance in partnership with University of Oxford and Wellcome Sanger Institute) and African CDC Pathogen Genomics Initiative (https://ipg.africacdc.org/).

b) Pooling and sequencing: Please explain how pooling will be done and how you will keep enough biological diversity to avoid missing rare ARGs.

Response: Pooling will be undertaken across reservoirs (page 7), i.e. pooling samples from each environmental source and from healthy humans by village, age, and season; pooling hospital samples by site, age and season. As our primary objective is to assess to what extent clinical AMR in invasive infections can be predicted, by age and by season, we are not really focusing on catching rare ARGs, and we acknowledge we may miss some rare ones due to sample size and the sequencing depth selected.

c) Validation: Describe how metagenomic data will be compared with hospital isolates and what measures (e.g., correlation, AUC) will be used to test accuracy.

Response: Please see earlier description of the model in the response to reviewer 1, point b. We will evaluate multiple Bayesian hierarchical binomial models constructed using different taxonomy-adjusted AMR metrics to predict the proportion of resistant invasive isolates. Model performance will be compared using Bayesian leave-one-out cross-validation (LOO-CV), which

estimates pointwise out-of-sample predictive accuracy and penalises model overfitting. Baseline models including only resistance gene abundance metrics, only taxonomic metrics, and an intercept-only null model will also be assessed to determine the added value of combining taxonomic and AMR data. The within-sample fit of the selected model will be evaluated using logarithmic scoring of posterior predictions against observed resistance counts, providing a principled measure of predictive quality based on information theory.

d) Sampling: Clarify how villages were chosen and whether this selection ensures diverse settings.

Response: In both Burkina Faso and Kenya, eight villages will be purposely selected from within the areas covered by each Health Demographic Surveillance System (HDSS) in Nanoro and Kilifi respectively. Selection will be made from the HDSS catchment areas, following geographical stratification to ensure representing the range of population, geographic and environmental diversity. Household GPS coordinates will be available in both Burkina Faso and Kenya. Only villages with at least one provider of antibiotics are eligible (see page 6)

e) AMR gene interpretation: Briefly describe how you will link ARGs to their host organisms or functions to improve biological meaning.

Response: While culturing data is often considered gold standard for linking ARGs to host organisms, this can also (to an extent) be done with the assembly based analysis that allows to determine the genetic context of ARGs. More specifically, linking ARGs to host organisms is challenging with short-read sequencing, but we will explore this by undertaking metagenomic assembly and considering the relative abundances of species and ARG taxa. In terms of functional annotation, in this project we are primarily interested in ARGs, but we will make our sequencing data publicly available to ensure other researchers to explore functions annotation in greater detail. We have added this in the *metagenomic sequencing and profiling* section, page 7-8.

f) How will differences between metagenomic and culture-based AMR results be interpreted?

Response: We would like to clarify that we will not be in a position to add culturing to our protocol. The current capacity of the local laboratories does not allow this, and there are no additional resources available.

g) Will host or immune-related data be collected for context?

Response: For context, data will be collected on anthropometry, WASH indicators, animal contact, and health care seeking behaviour.

h) What controls will ensure quality and reproducibility between labs?

Response: Standardised SOPs for sample storage, DNA extraction and extract transport to the sequencing facility in Basel have been established at the sites; pilots have demonstrated feasibility of the methods being used in generating metagenomic sequencing data. Prior to shipment of extracts, basic DNA quantity and quality (Nanodrop) readings will be shared with Basel. In Basel, further qualit control of extracts will be done (e.g. Qubit) prior to sequencing.

i) How will seasonal changes affect AMR results and how will this be analyzed?

Response: Seasonality may indeed impact on AMR, that is why we will collect similar data in the same villages once during the dry season, and once during the wet season. To account for potential seasonal variation in gene abundance and subsequent AMR resistance patterns in

clinical isolates, we will first describe seasonal differences in metagenomic resistome composition and abundance metrics descriptively. Following these analyses, we will include season (wet vs dry) as a covariate in the Bayesian hierarchical binomial models used to predict clinical resistance, and test an interaction between season and metagenomic predictors (e.g., taxonomy-adjusted AMR metrics) to assess whether associations differ by season. In addition, we will fit season-stratified Bayesian models to evaluate whether model coefficients and predictive accuracy (based on leave-one-out cross-validation, LOO-CV) differ between seasons. Posterior predictive checks will confirm that the models capture observed seasonal patterns, and environmental covariates such as rainfall will be incorporated where available. We have added this on page 9.

j) How will sequencing cost and pool size be optimized for future use?

Response: We are considering two different pool sizes and are exploring pooling of faecal samples prior to DNA extraction versus individual extractions followed by pooling of extracts. If our approach is validated to be effective in predicting resistance in clinical isolates in this study, we aim to explore a cost-utility analysis comparing different strategies for sentinel surveillance surveys based on metagenomics data, including detailed costings of the different strategies and resistance predictions obtained using models obtained as we vary i) sequencing depth; ii) number of samples per population-level cluster; iii) individual sample DNA extractions versus DNA extraction from pooled samples and (iv) sequencing costs. This analysis, which will be carried out within a Bayesian framework, will answer the question: under what circumstances will metagenomics-based sentinel surveillance be cost-effective compared with no surveillance as a result of improved empirical antibiotic prescribing. We will use simulations to explore how cost-utility varies with different sampling strategies in the surveillance data (including varying environmental sample pool sizes), varying AMR prevalence and the degree of geographic heterogeneity within the population of interest. This work will directly address the question of how such a surveillance system should be deployed at a national or regional level, what the cost implications would be, what the potential benefits to human health would be when using such data to inform antibiotic policies, and under what circumstances such an intervention would be considered cost-effective under different assumptions about willingness to pay for health benefits. This is now further explained under *statistical analysis*, page 8, and *cost-utility*, page 9.
